# RETRACTED: Hu et al. Hybrid Application of Nanoparticles and Polymer in Enhanced Oil Recovery Processes. *Polymers* 2021, *13*, 1414

**DOI:** 10.3390/polym16192704

**Published:** 2024-09-25

**Authors:** Yanqiu Hu, Zeyuan Zhao, Huijie Dong, Maria Vladimirovna Mikhailova, Afshin Davarpanah

**Affiliations:** 1 The Pharmaceutical College of Jiamusi University, Jiamusi University, Jiamusi 154007, China; zhaozeyuan@jmsu.edu.cn (Z.Z.); donghuijie@jmsu.edu.cn (H.D.); 2 Department of Prosthetic Dentistry, Sechenov First Moscow State Medical University, 119992 Moscow, Russia; sfmsmu@mail.ru; 3 Department of Mathematics, Aberystwyth University, Aberystwyth SY23 3BZ, UK

The journal retracts the article titled “Hybrid Application of Nanoparticles and Polymer in Enhanced Oil Recovery Processes” [1], cited above.

Following the publication, concerns have been brought to the attention of the Editorial Office relating to the accuracy of the authorship, the validity of the findings, and relevancy of a significant number of citations included within this article [1].

Adhering to our complaint procedure, an investigation was conducted by the Editorial Office and the Editorial Board which was unable to fully verify the accuracy of the originally listed authorship list. Furthermore, the Editorial Board detected irregularities within the findings and confirmed the presence of a high number of citations that lacked sufficiently relevant information for this publication.

As a result, the Editorial Board has lost confidence in the integrity of the findings and decided to retract this publication [1], as per MDPI’s retraction policy (https://www.mdpi.com/ethics#_bookmark30 accessed on 23 September 2024). 

This retraction was approved by the Editor-in-Chief of *Polymers*.

The authors did not provide a comment on this decision.
